# Partial Order Reduction for Deep Bug Finding in Synchronous Hardware

**DOI:** 10.1007/978-3-030-45190-5_20

**Published:** 2020-03-13

**Authors:** Makai Mann, Clark Barrett

**Affiliations:** 8 Johannes Kepler University, Linz, Austria; 9 University of Birmingham, Birmingham, UK; Stanford University, Stanford, CA 94305 USA

## Abstract

Symbolic model checking has become an important part of the verification flow in industrial hardware design. However, its use is still limited due to scaling issues. One way to address this is to exploit the large amounts of symmetry present in many real world designs. In this paper, we adapt partial order reduction for bounded model checking of synchronous hardware and introduce a novel technique that makes partial order reduction practical in this new domain. These approaches are largely automatic, requiring only minimal manual effort. We evaluate our technique on open-source and commercial packet mover circuits – designs containing FIFOs and arbiters.

## 1 Introduction

Modern society relies increasingly on electronic systems, powered by hardware components that continue to grow in complexity and variety. Ensuring the functional correctness of these components is essential, as bugs and errors can have consequences ranging from undermining a company's reputation to jeopardizing human safety [1,22,25,32,33]. Most electronic designs must therefore include a significant verification effort, and this effort often consumes more time and resources than all other aspects of the design process [17,34].

Formal methods such as symbolic model checking have become a crucial part of the verification effort because of their strong guarantees and automation [24]. However, due to the *state space explosion problem* [14], model checking typically only works well for small- to medium-sized circuits with primarily control logic, limiting its potential for addressing industry verification challenges.

One approach for combating the state space explosion problem is *partial order reduction* [14]. While symbolic partial order reduction has been successfully applied for the verification of asynchronous systems [37], its use in synchronous systems has been limited. In this paper, we introduce a novel approach for adapting symbolic partial order reduction to model checking of synchronous hardware

⋆ This work was supported by the National Science Foundation Graduate Research Fellowship Program under Grant No. DGE-1656518. Any opinions, findings, and conclusions or recommendations expressed in this material are those of the author(s) and do not necessarily reflect the views of the National Science Foundation. This work was also supported by the Defense Advanced Research Projects Agency, grant FA8650-18-2-7854. We thank the funding agencies and our corporate collaborators for their support.

A. Biere and D. Parker (Eds.): TACAS 2020, LNCS 12078, pp. 367–386, 2020.
https://doi.org/10.1007/978-3-030-45190-5_20

and demonstrate dramatic reductions in the time to reach deep bugs on certain classes of synchronous circuits. Moreover, the technique requires only an interface-level annotation of the circuit, and when fully automated approaches fail, can be guided by the user. The paper makes the following contributions:

1. We adapt partial order reduction for synchronous hardware verification.
2. We introduce a novel technique for reducing the possible inputs to a circuit at a single time step, which is crucial for practical application of partial order reduction to synchronous hardware.
3. We provide a set of sufficient conditions, which, if proven, guarantee that the proposed techniques maintain the reachable states.
4. We introduce conservative proof techniques for verifying these conditions, which empirically work well on packet movers.
5. We evaluate our techniques on a set of open-source and commercial packet mover circuits, demonstrating dramatic speed-ups with minimal manual effort.

The rest of the paper is organized as follows. We first provide a motivating example, below. Then, in Section 2, we cover relevant background material and notation. We explain our partial order reduction in Section 3 and our interface simplification technique in Section 4. We provide an experimental evaluation in Section 5. Section 6 covers related work, and Section 7 concludes.

### 1.1 Motivating Example

Throughout this paper we use the running example shown in Code Snippet 1. We chose this example because: i) it is easy to understand; ii) it resembles real-world packet mover circuits; and iii) it contains a difficult to reach bug.

The system has a synchronizing clock and takes two 1-bit inputs: `inc_x` and `inc_y`. The 6-bit registers (state elements) `x` and `y` index the `valid` vector and are initialized to 0. The 64-bit registers `valid` and `data` start at 0 and 1, respectively. The 64x64 bit memory is uninitialized. If `inc_x` and `en_x` are true, the system increments the value of `x`. When `inc_y` is true, the system increments `y`, sets the valid bit at index `y`, writes `data` to the memory at location y, and rotates the `data` vector to the left. Notice that the `en_x` signal ensures that `x` never surpasses `y` (until all bits in `valid` are set). This incrementing pointer logic is similar to that found in a circular pointer FIFO. To ensure the asserted property, the code attempts to maintain the invariant: `data = 1 << y`.

At first, it appears that the asserted property should hold based on this invariant, but it does not. There is a bug that can first occur at cycle 65: the overflow check in the `data` update uses integers, which are assumed to be 32-bits. Since `y` is zero-extended to be 32-bits, `y+1` can never be equal to 0. Thus, when `y` has the value 63 and is incremented, `data`, which is supposed to be one-hot, is set to 0.

Although the system is small, this is a surprisingly difficult bug to reach using model checking. We believe this is due in part to the non-determinism in the

Code Snippet 1: Buggy Toy Example

```
module deep_bug(input clk, input inc_x, input inc_y);
   reg [5:0]        x = 0;
   reg [5:0]        y = 0;
   reg [63:0]       valid = 0;
   reg [63:0]       data = 1;
   reg [63:0]       mem [63:0];
   wire             en_x;
   assign en_x      = valid[x]; always @(posedge clk) begin
      if (inc_x & en_x)
         x <= x + 1;
      if (inc_y) begin
         y <= y + 1; valid[y] <= 1'b1; mem[y] <= data;
         data <= (y+1 == 0) ? 1 : (data << 1);
      end
   end always @*
      assert ((mem[x] == (1 << x)) || ~valid[x]);
endmodule // deep_bug
```

update logic. In every state, there are 4 possible input combinations. As a result, there are an exponential number of execution paths. Model checkers routinely verify hardware designs with an exponential number of reachable states; however, we have observed that systems such as this which also have an exponential number of execution paths are difficult for a model checker to manage.

Specifically, all but two of the model checker configurations we tried timed out at 2 hours before reaching the bug. Since bounded model checking (BMC) is one of the best approaches for bug-finding, we focus on improvements to BMC that help reach this bug. We introduce automated, best effort techniques that reduce the time to hit this bug from over 1000 seconds to 46 seconds by safely adding temporal symmetry breaking constraints to the system.

## 2 Background

Before explaining our algorithm, we adapt the standard notion of synchronous transition systems and review fundamental model checking concepts below. For a more thorough introduction to model checking, we refer the reader to [14,15].

**Definition 1.** *A Synchronous Transition System (STS) is a tuple,* $\langle S, Init, A, En, D, T\rangle$*:*

- $S$*: a set of states*
- $Init \subseteq S$*: a set of initial states*

- *A: a finite set of atomic actions - logically distinct operations of the system*
- $En = \{en_a | a \in A\}$*: where* $en_a : S \to \mathbb{B}$ *is a state predicate that holds* iff *action a is* enabled *in a given state*
- *D: a set of data inputs to the system*
- $T \subseteq S \times (\mathcal{P}(A) \times D) \times S$*: the state transition relation, where* $\mathcal{P}$ *denotes power set*

For our purposes, an STS instruction can perform multiple atomic actions simultaneously. We define the system's *instruction set* (i.e. the set of actions that the system can perform in one transition) as $\mathcal{I} := \mathcal{P}(A)$. We then define the set of *inputs* of an STS as $Input := \mathcal{I} \times D$. Thus, the transition relation $T$ is a subset of $S \times Input \times S$.

We denote the cardinality of an instruction $i$ as $|i|$. For $s, s' \in S, in \in Input$, $T(s, in, s')$ holds *iff* it is possible to reach $s'$ from $s$ by applying input $in$. It is often convenient to reason about sequences using vector notation. Let $\boldsymbol{in} \in Input^n$ and $\boldsymbol{s} \in S^{n+1}$, with $n > 0$. We use subscripts to name individual elements of vectors, e.g. $\boldsymbol{s} := \langle s_0, s_1, \dots \rangle$. We use the notation $T(\boldsymbol{in}, \boldsymbol{s})$ to denote $\bigwedge_{0 \leq i < n} T(s_i, in_i, s_{i+1})$. The length of a vector is given by $| \cdot |$, e.g. $|\boldsymbol{s}| = n + 1$, and prepending is represented as $\cdot : \cdot$, e.g. $\boldsymbol{s} = s_0 : \boldsymbol{s'}$ for some $s' \in S^n$. With some abuse of notation, we allow prepending both sequences and single elements. For $k > 0$, we say that $\boldsymbol{s} \in S^k$ is reachable if $\exists n \in \mathbb{N}, \boldsymbol{s'} \in S^{n+1}, \boldsymbol{in} \in Input^{n+k} \ . \ Init(s'_0) \land T(\boldsymbol{in}, \boldsymbol{s'} : \boldsymbol{s})$.

The set of enabledness predicates $En$ constrain the valid states in which an action can occur. For an instruction $i \in \mathcal{I}$ and $s \in S$, let $en_i(s) := \bigwedge_{a \in i} en_a(s)$. In the remainder of the paper, we only consider transition relations $T$ that respect the enabledness conditions. That is, we assume $\forall\, s, i.(en_i(s) \leftrightarrow \exists\, s', d.T(s, \langle i, d \rangle, s'))$. Depending on the context, this can be checked with a model checker or added as an environmental assumption. We also assume that the existence of a transition does not depend on the data input, that is, $\forall\, s, i.\, (\exists\, d, s'.\, T(s, \langle i, d \rangle, s') \implies \forall\, d.\, \exists\, s'.\, T(s, \langle i, d \rangle, s'))$.

*Example 1.* We can define an STS for the motivating example. Let $BV_k$ denote the set of all bitvectors of width $k$. Because there is only a single clock with no negative edge behavior, we model the system without the clock, where every transition corresponds to a clock cycle. Define an STS $\langle S, Init, A, En, D, T \rangle$, where:

- $S = BV_6 \times BV_6 \times BV_{64} \times BV_{64} \times (BV_{64})^{64}$ is the set of values for $\langle \text{x}, \text{y}, \text{valid}, \text{data}, \text{mem} \rangle$
- *Init* is the set containing all states where x = 0, y = 0, valid = 0 and data = 1
- $A = \{\text{inc\_x}, \text{inc\_y}\}$
- $En = \{en_{\text{inc\_x}} := \text{valid}[x] = 1, en_{\text{inc\_y}} := true\}$
- $D = \{nil\}$ (here, *nil* is just a dummy placeholder used to ensure that $T$ is not empty).
- $T$ is the relation describing the next state updates in Code Snippet 1.

**Model Checking.** Given an STS $\mathbf{S}$, let a safety property $P \subseteq S$ be a set containing acceptable states. The *model checking problem* is to determine whether the system stays within this acceptable set for all possible execution traces. Formally, we want to check whether the following holds:

$$\forall\, n \geq 0, \boldsymbol{in} \in \mathit{Input}^n, \boldsymbol{s} \in S^{n+1}.\, (\mathit{Init}(s_0) \wedge T(\boldsymbol{in}, \boldsymbol{s})) \implies s_n \in P \qquad (1)$$

When equation (1) holds, we say that $P$ is an invariant of $\mathbf{S}$. A number of techniques exist for solving this problem, including Binary Decision Diagram (BDD)-based [12] approaches, Interpolant-based [27] approaches, and IC3/PDR (property directed reachability) techniques [10,16]. We refer the interested reader to [15] for a more complete survey of model checking algorithms.

In this paper, we will focus on *bounded model checking* (BMC). In BMC, instead of proving (1) for all $n$, we prove it for all $n$ less than some finite bound $k$. Though it typically cannot be used to prove properties, BMC can be quite effective at finding bugs [6] and is especially useful when full model checking is infeasible.

**Symmetry.** Early on in the development of model checking, researchers recognized the importance of symmetry reduction to combat the state explosion problem [13]. Existing approaches in the hardware domain perform data symmetry reduction and data type reduction through the use of bit-width reduction preprocessing passes or syntactic restrictions such as *scalarsets* [8,20,28]. There have also been abstraction-refinement loop algorithms proposed to handle memory symmetries [9]. All of these approaches are focused on symmetries present in the transition system description, such as the presence of large data types. We refer to these types of symmetries as *data* symmetries. Most of these techniques are intended to speed up proofs of true properties rather than accelerate bug-finding.

Model checking of asynchronous systems such as concurrent programs faces an orthogonal issue due to the many possible redundant interleavings of independent processes. Throughout this paper, we refer to this as *path* symmetry. Path symmetry is a temporal symmetry: it relates to executions of a system rather than just its size. Path symmetries occur when there are many distinct ways of reaching the same state in a system execution. Exploring all such paths can result in exponential case splitting.

This paper provides evidence that path symmetry can also severely hurt model checking performance in synchronous systems. One of the first techniques proposed to handle path symmetry was partial order reduction.

**Partial Order Reduction.** Partial order reduction was first developed in the explicit-state model checking context but was later extended to symbolic model checking [37]. The approach is named “partial order reduction” for historical reasons, but Clarke noted in [14] that “model checking using representatives” [30,31] may have been a more appropriate name. In particular, partial order reduction attempts to develop equivalence classes of behaviors so that only one representative from each class needs to be considered during model checking. Note that partial order reductions are sound only for checking state invariants. If the property of interest is temporal, the reduction could disallow input sequences that trigger the property. This can be avoided by first instantiating a monitor [15] and, if necessary, converting liveness properties to safety [5].

Partial order reduction is less natural in the synchronous setting, because synchronous transition systems do not have easily expressible independent actions. Nevertheless, these systems can still benefit from partial order reduction. Consider our motivating example: despite the huge number of system execution paths to consider, many of them are redundant. Observe that if both inputs are zero, then the state does not change. Furthermore, there is a temporal symmetry in the system execution: from any state where `en_x` is true, driving only `inc_x` followed by only `inc_y` results in the same state as driving them in the opposite order. Thus, this system has a large number of redundant interleavings, much like a multi-threaded program. To address this problem, we introduce a partial order reduction for synchronous hardware. Our goal is to remove redundant interleavings by adding constraints to the system. To maintain soundness, we provide a set of conditions which must pass before we can add constraints.

## 3 Synchronous Partial Order Reduction

In order to be able to apply partial order reduction to a synchronous transition system, we are interested in identifying pairs of instructions that can be reordered without affecting the resulting state. More generally, we also want to be able to find pairs that can only be reordered under certain conditions. To formalize these notions, we adapt the notation and representation of *guarded independence relations* from [37].[1]

**Definition 2.** *Given an STS: $\langle S, Init, A, En, D, T\rangle$ with instruction set $\mathcal{I}$, let $G := \mathcal{P}(S)$ be the set of predicates over the states. Let $\langle i_0, i_1, g\rangle$ be a* guarded independence tuple *iff for all $d_0, d_1 \in D$ and reachable $\boldsymbol{s} \in S^3$, the following condition holds:*

$$en_{i_0}(s_0) \wedge g(s_0) \wedge T(\langle\langle i_1, d_1\rangle, \langle i_0, d_0\rangle\rangle, \boldsymbol{s}) \implies \exists\, s'.T(\langle\langle i_0, d_0\rangle, \langle i_1, d_1\rangle\rangle, \langle s_0, s', s_2\rangle).$$

According to this definition, if we can prove that $\langle i_0, i_1, g\rangle$ is a guarded independence tuple, then we can reorder $\langle i_1, i_0\rangle$ instruction sequences as long as i) $i_0$ is enabled in the first state; ii) $g$ holds in the first state; and iii) we also reorder the corresponding data inputs. We check only the enabledness of $i_0$ because $\langle i_0, i_1\rangle$ is the representative order, and we only need to be able to reorder to the representative, not from it. The guard allows us to consider partial order reductions that only hold for a subset of the reachable states. To avoid trivially overconstraining the system with conflicting reorderings, we will only consider one ordering for each pair of instructions.

The condition in Definition 2 is difficult to check automatically because of the existential quantifier. We instead check two slightly weaker conditions that

[1] The main differences are our STS formalism and that we consider reachability.

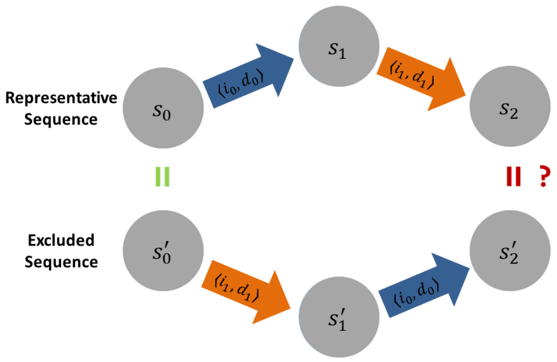

Fig. 1: Partial Order Reduction Condition (3) Proof Goal imply guarded independence. These conditions are also standard in the POR literature [14,37]. The first condition states that instruction $i_0$ cannot disable $i_1$ under guard $g$:

$$\forall\, d \in D, \boldsymbol{s} \in S^2.\ (en_{i_1}(s_0) \wedge g(s_0) \wedge T(s_0, \langle i_0, d\rangle, s_1)) \implies en_{i_1}(s_1) \tag{2}$$

Intuitively, this condition ensures that we do not remove reachable states by disabling instructions. The second condition is that executing the instructions in either order leads to the same final state:

$$\begin{aligned}&\forall d_0, d_1 \in D, \boldsymbol{s}, \boldsymbol{s'} \in S^3\ .\ (g(s_0) \wedge (s_0 = s'_0) \wedge \\ &T(\langle\langle i_0, d_0\rangle, \langle i_1, d_1\rangle\rangle, \boldsymbol{s}) \wedge T(\langle\langle i_1, d_1\rangle, \langle i_0, d_0\rangle\rangle, \boldsymbol{s'})) \implies (s_2 = s'_2)\end{aligned} \tag{3}$$

When applying partial order reduction to concurrent programs, the standard approach is to check conservative syntactic properties which guarantee conditions (2) and (3). Synchronous systems do not typically have these syntactic properties, because there is no notion of distinct processes. Instead, we must check these conditions directly. In real circuits, it is unlikely that (2) will hold over arbitrary states. However, it is sufficient to prove that it holds for all reachable states. This can be done with a model checker.

To prove (3), we could encode it as an LTL property or build a monitor automaton and use a model checker. Alternatively, we have found that we can often use a straightforward commuting-diagram approach starting from a symbolic initial state, depicted in Fig. 1. We duplicate the system, unroll it twice, then start both copies in the same symbolic state and check that applying the instructions in either order results in the same final state. This simple approach has the disadvantage that a symbolic initial state ignores reachability which could lead to spurious counterexamples. However, notice that the initial state is constrained by enabledness assumptions. To apply an instruction it must be enabled, so both instructions must be enabled in the initial state. We have found that these enabledness assumptions often constrain the initial state enough to rule out spurious counterexamples.

If both conditions pass, then we can choose a representative order and disallow the opposite ordering for that pair of instructions. If the proof of condition (3) fails, it provides a counterexample which should either convince the user that partial order reduction does not apply for that pair of instructions (a real counterexample), or serve as a guide for the user to write guards that would remove the spurious counterexample. Other invariants of the system, either obtained automatically or manually guessed by the user, could also remove spurious counterexamples. We can now state the first theorem of synchronous partial order reduction: that these conditions guarantee guarded independence over all reachable states.

**Theorem 1.** *Given an STS* $\mathbf{S} := \langle S, \mathit{Init}, A, \mathit{En}, D, T\rangle$*, with instruction set* $I := \mathcal{P}(A)$*: if conditions* (2) *and* (3) *hold for instructions* $i_o, i_1 \in I$*, and guard* $g \in \mathcal{P}(S)$*, then* $\langle i_0, i_1, g\rangle$ *is a guarded independence tuple.*

*Proof.* Assume conditions (2) and (3) and that for some $d_0, d_1 \in D$ and reachable $\boldsymbol{s} \in S^3$, we have:

$$en_{i_0}(s_0) \wedge g(s_0) \wedge T(\langle\langle i_1, d_1\rangle, \langle i_0, d_0\rangle\rangle, \boldsymbol{s})$$

Because $en_{i_0}(s_0)$, we have $\exists s', d' \,.\, T(s_0, \langle i_0, d'\rangle, s')$ because of our enabledness assumption. Furthermore, by the data-input independence property of transition relations, it follows that for some $s'_1$, $T(s_0, \langle i_0, d_0\rangle, s'_1)$ Now, because one of our assumptions is a transition from $s_0$ using $i_1$, $en_{i_1}(s_0)$ must be true. Condition (2) implies that $en_{i_1}(s'_1)$, thus $\exists\, s', d'.\, T(\langle\langle i_0, d_0\rangle, \langle i_1, d'\rangle\rangle, \langle s_0, s'_1, s'\rangle)$. As before, this implies that for some $s'_2$, we also have that $T(\langle\langle i_0, d_0\rangle, \langle i_1, d_1\rangle\rangle, \langle s_0, s'_1, s'_2\rangle)$. It then follows from (3) that $s'_2 = s_2$, and thus, $\langle i_0, i_1, g\rangle$ satisfies the condition from Definition 2. ☐

Let a guarded independence relation, $R \subseteq \mathcal{I} \times \mathcal{I} \times G$, be a set of guarded independence tuples. We now describe how to apply partial order reductions, given some $R$. For each $\langle i_0, i_1, g\rangle \in R$, and for every $\boldsymbol{s} \in S^2, d \in D$, whenever $T(s_0, \langle i_1, d_1\rangle, s_1) \wedge en_{i_0}(s_0) \wedge g(s_0)$ holds, we remove from $T$ every transition of the form $\langle s_1, \langle i_0, d\rangle, s\rangle$ (for any $d$ and $s$). Let $T^R$ be the result. To apply this reduction in practice, we add a constraint to the BMC encoding: $(g(s_0) \wedge en_{i_0}(s_0) \wedge i_1) \implies \neg \mathit{next}(i_0)$.

This makes it impossible for the STS system to ever execute an instruction $i_0$ after an instruction $i_1$ when starting from a state where $i_0$ is enabled and $g$ holds. This effectively gives preference to $i_0$ as long as it is enabled. The effect of partial order reduction on a pair of instructions in a synchronous system is depicted in Fig. 2. Red X's show removed transitions, and for simplicity, we assume a trivial guard of *true*. Notice that all states are still reachable via some path from the initial state in the bottom left corner.

**Theorem 2.** *Given* $\mathbf{S} := \langle S, \mathit{Init}, A, \mathit{En}, D, T\rangle$*, let* $R$ *be a guarded independence relation and let* $\mathbf{S}_R$ *be the* reduced *STS obtained by replacing* $T$ *with* $T^R$ *in* $\mathbf{S}$*. Then, if a property* $P$ *is an invariant for* $\mathbf{S}_R$*, it is also an invariant for* $\mathbf{S}$*.*

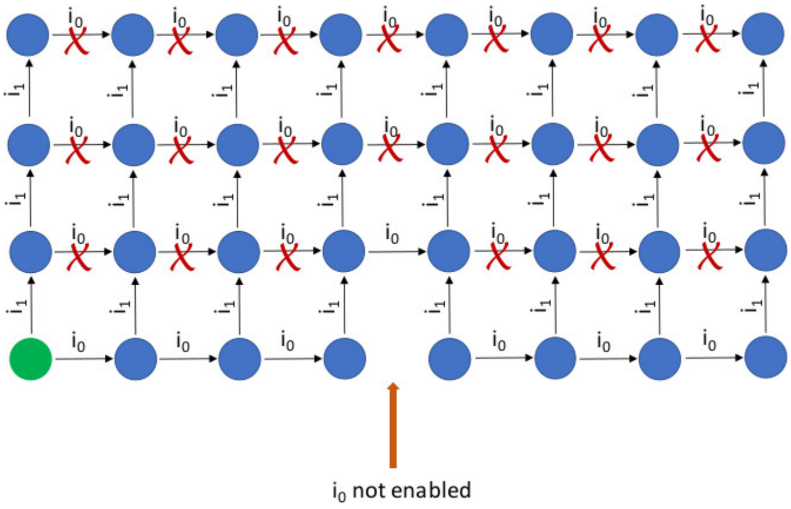

Fig. 2: Effect of Partial Order Reduction for Instructions $i_0$ and $i_1$. Initial state is green.

*Proof.* It suffices to show that $\mathbf{S}_R$ can reach all the same states as $\mathbf{S}$. We prove this by contradiction. Assume there is some $\boldsymbol{in}, \boldsymbol{s}$ such that $Init(s_0) \wedge T(\boldsymbol{in}, \boldsymbol{s})$ and $0 \leq j \leq |\boldsymbol{s}| - 1$ such that $s_j$ is the first state that is unreachable in $S_R$. The value of $j$ cannot be 0 or 1, because $\mathbf{S}$ and $\mathbf{S}_R$ have the same initial states and $T^R$ only excludes sequences of length 2. Then, by the definition of $T_R$, $\langle in_{j-2}, in_{j-1} \rangle$ must be a sequence excluded by $T^R$. Conditions (2) and (3) guarantee that permuting $in_{j-2}$ and $in_{j-1}$ results in an enabled sequence that ends in the same state, $s_j$, which contradicts the assumption. Thus, there cannot be a state which is reachable in $\mathbf{S}$ but not $\mathbf{S}_R$. □

## 4 Reduced Instruction Sets

Now that we can apply partial order reduction to synchronous systems, our main goal is to identify a maximal guarded independence relation, $R$. Recall that we defined instructions as sets of atomic actions. We call an instruction containing at most one action *atomic* (this includes the instruction with *no* actions). Non-atomic instructions are *complex*. Instructions thus reflect the parallelism of synchronous hardware, and lead to natural candidates for $R$: pairs of atomic instructions.

Furthermore, notice that the number of instructions is exponential in the number of actions. Thus, it could be prohibitively expensive to check every pair of instructions for guarded independence. In contrast, the number of *atomic* instructions is equal to the number of actions (plus one). Furthermore, it is likely that many complex instruction pairs will not have a guarded independence relationship because they contain common actions. Our goal in this section is to disallow as many complex instructions as possible without losing any reachable states, thereby reducing the number of pairs of instructions we need to check while also making it more likely for the checks to succeed. Note that, in isolation, removing instructions might be problematic, because it could extend the bound needed to reach a property violation. However, as we will demonstrate in the experimental section, this disadvantage is more than compensated for when it is applied in combination with partial order reduction.

Given an STS with instruction set $\mathcal{I}$, we seek a reduced instruction set, $\mathcal{I}_r \subseteq \mathcal{I}$, which preserves the reachable states of the system. Let $\mathit{Input}_r$ be the set of inputs which only use instructions from $\mathcal{I}_r$. Given an input $\mathit{in} \in \mathit{Input}$, our goal is to prove the existence of a witness $w(\mathit{in}) \in \mathit{Input}_r^n$ (for some $n > 0$) that simulates the behavior of $\mathit{in}$ using only reduced instructions. Formally, the witness function $w$ should satisfy:

$$\begin{aligned} &\forall\, s, s' \in S, \mathit{in} \in \mathit{Input}.\, T(s, \mathit{in}, s') \implies \\ &\quad \exists\, n \in \mathbb{N}, \boldsymbol{s} \in S^n.\, T(w(\mathit{in}), s : \boldsymbol{s}) \wedge (s_{n-1} = s') \end{aligned} \tag{4}$$

In other words, we need to show that for every instruction in the original instruction set, there exists a sequence of inputs, using only instructions from the reduced instruction set (RIS), that results in the same final state. Notice that a witness function that also depended on the state would be more general, but for our purposes, it is sufficient for the witness function to depend only on the input.

### 4.1 Atomic instruction sets

The condition in (4) is quite general and does not provide any intuition on how to choose $w$. Here, we focus on a specific case where $w$ is easy to construct: we choose $\mathcal{I}_r$ to be an *atomic instruction set*, defined as an instruction set containing only atomic instructions. We then must prove that the set of reachable states is not affected by restricting the instructions to those in $\mathcal{I}_r$.

It is sufficient to prove that for each complex instruction, we can remove one of its actions and perform that action in the next step, with the same result. For some complex instruction $i$ containing $a$ and some data input $d$, let $w^a(\langle i, d\rangle)$ be $\langle\langle i - \{a\}, d\rangle, \langle\{a\}, d\rangle\rangle$. We must show that for each input $\mathit{in}$ containing a complex instruction, there exists some $a$ where $w^a(\mathit{in})$ has the equivalent effect on the system as $\mathit{in}$. Formally, the requirement is:

$$\begin{aligned} &\forall i \in \mathcal{I} \setminus \mathcal{I}_r, d \in D, \boldsymbol{s} \in S^2. \\ &\quad T(s_0, \langle i, d\rangle, s_1) \implies \exists\, a \in i, \boldsymbol{s'} \in S^3.\, T(w^a(\langle i, d\rangle), \boldsymbol{s'}) \wedge s_0 = s'_0 \wedge s_1 = s'_2 \end{aligned} \tag{5}$$

Condition (5) is still difficult to prove because of the existential quantifier. One conservative approach is to replace the existential quantifier with a universal quantifier and attempt to prove that stronger condition. For real systems, this is unlikely to hold. Instead, we propose a counterexample blocking procedure which, if it succeeds, guarantees (5). We introduce symbolic values for $i$, $d$, and $a$ and then iteratively add constraints over them until the proof succeeds or

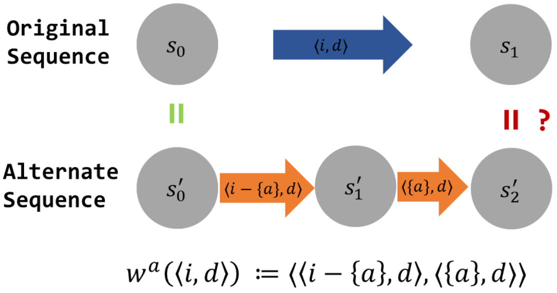

Fig. 3: Equivalence of original and reduced instruction sequences. Circles represent states.

we have enumerated all possibilities. This algorithm is a specialized $\forall\exists$ decision procedure that exploits the structure of (5) and additional domain knowledge about the proof goal. We use a constraint solver as an oracle.

**Algorithm 1 ProveRIS(S)**

$\mathbf{S}' := \langle S', Init', A', En', D', T'\rangle \leftarrow copy\_sys(\mathbf{S})$
$\mathcal{I} := \mathcal{P}(A)$, $\mathcal{I}' := \mathcal{P}(A')$ // instruction sets are power sets of actions
**var** $i : \mathcal{I}$, **var** $i' : \mathcal{I}'$, **var** $a : A$
**var** $\boldsymbol{s} : S^2$, **var** $\boldsymbol{s}' : S'^3$, **var** $d : D$, **var** $d' : D'$
$add\_constraint(s_0 = s'_0 \wedge d = d' \wedge i' = i - \{a\})$
$add\_constraint(T(\langle\langle i, d\rangle\rangle, \boldsymbol{s}) \wedge T'(\langle\langle i', d'\rangle, \langle\{a\}, d'\rangle\rangle, \boldsymbol{s}'))$
**for** c = 2 ... $|A|$ **do**
  **while** $check\_sat(|i| = c \wedge s_1 \neq s'_2)$ **do**
    $\mu \leftarrow get\_model()$
    $i_\mu \leftarrow assignment(\mu, i)$
    $a_\mu \leftarrow assignment(\mu, a)$
    $add\_constraint(i_\mu \subseteq i \implies a \neq a_\mu)$
    **if** $\neg check\_sat(i = i_\mu)$ **then**
      **return** false // exhausted all possible decompositions for this instruction
    **end if**
  **end while**
**end for**
**return** true // every instruction can be decomposed

Algorithm 1 takes an STS, $\mathbf{S} := \langle S, Init, A, En, D, T\rangle$ and returns true if the instruction set can be decomposed into an atomic instruction set by delaying a single action from each instruction.[2] For simplicity, the algorithm assumes (and we check this assumption separately) that if a complex instruction $i$ is enabled, then for each $a \in i$, executing $i - \{a\}$ results in a state where $a$ is enabled.

[2] We also implemented a more general version of this algorithm which can drop more than one action at a time from the instruction $i$, but this simpler version is sufficient for the results we report in this paper.

Formally:

$$\forall\, i \in \mathcal{I} \setminus \mathcal{I}_r, d \in D, \boldsymbol{s} \in S^2, a \in i.\ en_i(s_0) \wedge T(s_0, \langle i - \{a\}, d\rangle, s_1) \implies en_a(s_1) \quad (6)$$

Note that this is only a slight generalization of the property that atomic instructions do not disable each other, a condition that we will need anyway in order to apply partial order reduction to the atomic instruction set (see condition (2)).

The algorithm first creates an identical copy of the STS in line 1. Lines 2-4 set up symbolic variables for the instructions, data, and states of each system. Line 5 adds constraints to the solver enforcing that both systems start in the same state, use the same data, and that $i'$ is $i$ but with symbolic action $a$ dropped. Line 6 adds the transition relation constraint for each STS. The initial symbolic set up is depicted in Fig. 3.

The outer loop at line 7 iterates over all possible complex instruction cardinalities. The inner loop starting at line 8 attempts to show that for each cardinality $c$, instructions of that cardinality can be decomposed by delaying one action (symbolically represented by $a$). If all instructions of cardinality $c$ have been decomposed, then the while loop condition is false and the outer loop continues. Otherwise, it gets variable assignments from the constraint solver in lines 9-11 and learns a constraint at line 13 that prevents this particular action, $a_\mu$, from being chosen for decomposition again. To ensure that we have not blocked all possible actions, there is an additional check at line 13, which returns false in the case that no action can be delayed for the current instruction.

Importantly, the algorithm assumes that if the delay of action $a_\mu$ does not create a valid witness sequence for a given complex instruction $i_\mu$, then the same is true *whenever* the instruction $i$ includes $i_\mu$. We call this a *monotonicity* assumption, and it typically holds when actions are somewhat independent. The monotonicity assumption motivated the current structure of the algorithm and can significantly reduce the number of iterations in the algorithm. We can remove this assumption by changing $i_\mu \subseteq i$ to $i_\mu = i$ in the antecedent in line 13. Note that the monotonicity assumption does not make the algorithm unsound: if it returns true, then (as we prove below) condition (5) holds. However, if the algorithm returns false, then it may be that the version without the assumption would return true. For each of our experiments, we were able to get a true result with the monotonicity assumption.

Because the algorithm does not consider state reachability and looks for a witness function that only depends on inputs, it can still return false when an equivalent sequence might exist for reachable states. In such cases, users can examine the constraint solver models and attempt to remove some of them by proving other invariants.[3]

If algorithm 1 returns true, we replace $T$ with $T_r$, where $T_r$ is the result of removing from $T$ all transitions $\langle s, \langle i, d\rangle, s'\rangle$ where $|i| > 1$. Practically, this is

[3] This was rarely necessary in our experiments. Our implementation also extended the algorithm to support predicate abstraction, which could also rule out spurious counterexamples, but this feature was never needed in our experiments.

achieved by adding a disjunctive constraint over the possible atomic actions. We can now state the main results for reduced instruction sets.

**Theorem 3.** *Let $S$ be an STS. If condition (6) holds and* ***ProveRIS(S)*** *returns true, then (5) holds.*

*Proof.* We maintain the loop invariant at line 8 that for every instruction $i'$, there is some action $a'$ such that $check\_sat(|i| = c \wedge i = i' \wedge a = a')$ is true. It's true initially for each $c$ by condition (6). Afterwards, the check on line 14 ensures that it is maintained. Furthermore, the check on line 9 ensures that when the while loop is exited, then any satisfying assignment for $check\_sat(|i| = c)$ is such that $s_1 = s'_2$. Together, these conditions guarantee that (5) holds.

**Theorem 4.** *Let $\mathbf{S} \coloneqq \langle S, Init, A, En, D, T\rangle$ be an STS such that condition (6) holds and* ***ProveRIS(S)*** *returns true, and let $T_r$ be the transition relation for the reduced instruction set. Let $\mathbf{S}_r$ be the* reduced *STS obtained by replacing $T$ with $T_r$ in $\mathbf{S}$. Then, safety property $P \in S$ is an invariant for $\mathbf{S}_r$ if and only if it is also an invariant for $\mathbf{S}$.*

*Proof.* It suffices to show that the reachable states of $\mathbf{S}$ and $\mathbf{S}_r$ are identical. *Init* does not change, so the initial states cannot be different. Furthermore, $T_r$ is obtained by removing transitions from $T$, we know that $S_r$ cannot add any reachable states. To show that it also does not remove any reachable states, consider an arbitrary trace $Init(s_0) \wedge T(\boldsymbol{in}, \boldsymbol{s})$ with $|s| = n$, we must show $\exists\, \boldsymbol{in'}, m, \boldsymbol{s'} \in S^m.\, Init(s'_0) \wedge T_r(\boldsymbol{in'}, \boldsymbol{s'}) \wedge s_{n-1} = s'_{m-1}$. We prove this by showing by induction that it holds whenever $\boldsymbol{in}$ contains instructions of cardinality at most $c$.

In the base case, $c = 1$, so all instructions are of size one or less. All of these are already atomic and thus we can take $\boldsymbol{in'} = \boldsymbol{in}$ and $\boldsymbol{s'} = \boldsymbol{s}$ by the definition of $T_r$.

For the inductive step, suppose that it holds for cardinalities up to $c-1$, and assume $Init(s_0) \wedge T(\boldsymbol{in}, \boldsymbol{s})$ with $|s| = n$. Let $in_j = \langle i, d\rangle$ be an input containing an instruction of size at most $c$. If $|i| < c$, there is nothing to be done. Thus we only consider the case where $|i| = c$. We know that $T(s_j, in_j, s_{j+1})$ holds. By Theorem 3 and condition (5), it follows that $T(\langle\langle i - \{a\}, d\rangle, \langle\{a\}, d\rangle\rangle, \langle s_j, s, s_{j+1}\rangle)$ holds for some $a$ and $s$. We can thus replace $in_j$ in $\boldsymbol{in}$ by $\langle i - \{a\}, d\rangle$ followed by $\langle\{a\}, d\rangle$ to obtain an input sequence $\boldsymbol{in}_c$ and insert $s$ between $s_j$ and $s_{j+1}$ in $\boldsymbol{s}$ to obtain $\boldsymbol{s}_c$ with final state $s_{n-1}$ such that $Init(s_0) \wedge T(\boldsymbol{in}_c, \boldsymbol{s}_c)$. Repeating this process for each input containing an instruction of size $c$ yields a final $\boldsymbol{in}_c$ such that the maximum cardinality of any instruction is $c-1$. The property then holds by the inductive hypothesis. □

Note that if there is some instruction $i \in \mathcal{I}$ which cannot be decomposed into atomic instructions, we could always keep this instruction in $\mathcal{I}_r$ and still benefit from removing other complex instructions. In many cases, we can also remove the empty instruction, $i_e = \emptyset$. If applying $i_e$ cannot change the state of the system, regardless of the data input, then it is considered a *stutter step* [14]. It is straightforward to check whether $i_e$ can be removed by comparing the state before and after applying $i_e$.

## 5 Experimental Results

We developed a prototype flow for proving the POR and RIS conditions and applying the necessary constraints. We use the IC3/PDR implementation in ABC [11], `pdr`, to prove condition (6) (which implies condition (2)). This requires manually writing a Verilog property for each atomic instruction.[4] We implemented the **ProveRIS** algorithm in our SMT-based model checker, CoSA [26], configured with boolector [29] on the `smtcomp19` branch, using CaDiCaL [4] as the underlying SAT solver.[5] We check the commuting diagram for condition (3) in CoSA as well. It tries the trivial guard `true` by default, and allows the user to provide additional candidate guards if necessary. The set up for proofs in CoSA is automated based on user-provided annotations for the actions and enable conditions. We show our best results which used an encoding leveraging the SMT theory of arrays to represent memories for proving conditions, and a pure bitvector encoding for bounded model checking.

Our flow applies the following steps: i) read in a system description in Verilog using Yosys [38] and generate AIGER [7] for ABC (or BTOR2 [29] for other tools); ii) check condition (6) for each atomic instruction; iii) run the **ProveRIS** algorithm, and if it returns true, add constraints to rule out all but atomic instructions; and iv) check POR condition (3) for each pair of atomic instructions and add constraints for each passing pair of instructions with the associated guard. Each step depends on the previous step passing successfully. In each of our experiments described below, we successfully completed every step of this flow, though in some cases guards were required in step (iv). For POR and RIS runtimes, we always include the time to check the conditions. We tried running with POR alone, but it resulted in negligible improvements in runtime and thus we omit these results. This demonstrates the importance of RIS. We ran all experiments on a 3.5GHz Intel Xeon CPU with 16GB of RAM.

### 5.1 Motivating Example

First, we return to our motivating example. We compare the time to reach the bug using the SAT-based ABC [11] engines `pdr` and `bmc`, and SMT-based bounded model checking using btormc [29] and CoSA. We ran the SMT-based model checkers both with and without the SMT theory of arrays for the encoding of the memory. Both btormc and CoSA without the array encoding were able to reach the bug in 1230s and 1437s, respectively, but all other approaches timed out at two hours. In particular, `pdr` times out at 2 hours on the property, but can

[4] This could be automated based on user-tagged actions and user-provided enable conditions.

[5] GitHub Commit Hashes for Tools:
Boolector/Btormc: 1989080261235f33e344cbd095e70a337c45bd16
CoSA: ff3c8cee1f0834c03167b2a8ecdd1223031312b3
PySMT: 09dc303185812149550110123ad266326beb1179
Yosys: a4b59de5d48a89ba5e1b46eb44877a91ceb6fa44
ABC: 5776ad07e7247993976bffed4802a5737c456782

| Design | # | #Solved | #Solved PR | Time | Time PR |
|---|---|---|---|---|---|
| com 1 | 49 | 35 | **47** | 103.8 | **20.3** |
| com 2 | 49 | 25 | **34** | 470.8 | **4.9** |
| cp | 49 | 35 | **47** | 230.3 | **18.9** |
| sr | 49 | 25 | **33** | 912.6 | **5.6** |
| arb n=2 | 49 | 35 | **42** | 89.1 | **20.5** |
| arb n=3 | 49 | 35 | **42** | 94.0 | **21.9** |
| arb n=4 | 49 | 35 | **42** | 101.3 | **35.5** |
| arb n=5 | 49 | 35 | **42** | 111.7 | **31.8** |

Table 1: Number Solved and Average Runtime

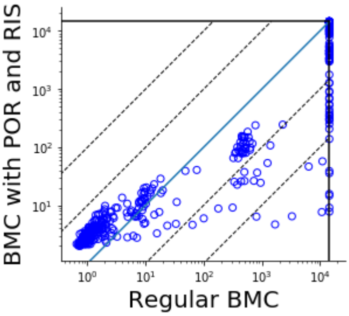

Fig. 4: Runtime Comparison prove condition (6) for every atomic instruction in less than a second. Intuitively, this makes sense because the enabledness conditions do not involve `data` or `mem`. Thus, none of the datapath falls in the cone of influence, leaving only control logic for IC3 to reason about. The remaining conditions, (3) and (5), are proven in less than three seconds. Since all the conditions pass, we apply the POR and RIS constraints, which reduces the time to hit the bug from 1437s to 46s in CoSA, including the time to check the conditions.

### 5.2 Packet Movers

We now evaluate our approach on data integrity properties for a variety of packet-mover circuits. Data integrity is a safety property that ensures no packets are dropped or corrupted. In practice, data integrity is often checked by instantiating a monitor, called a *scoreboard*. It provides the necessary infrastructure for formal verification. In our case, it non-deterministically tags a *magic packet* and checks that this packet exits the system when it should. Crucially, the scoreboard is a reusable module which can check data integrity of arbitrary packet movers.

Notice that existing symmetry reduction techniques will not be very effective for this scoreboard setup. For example, consider a circular pointer FIFO which maintains two incrementing pointers that index a memory for reading and writing, respectively. We cannot use scalarsets to break symmetries in the memory addresses because the pointers index the memory and are involved in arithmetic, breaking the syntactic requirements for scalarsets [28]. Furthermore, sequential memory abstraction [9] could reduce the size of the memory, but does not address the path symmetry. In addition, both these symmetry reduction techniques are focused on proofs, not bug-finding.

We evaluate our approach on two commercial library components from a major hardware company. We also implemented simpler, open-source versions of these designs. Our open-source benchmarks include: i) a circular pointer FIFO which assumes power-of-two depth but is instantiated with a non-power-of-two depth (one greater than the provided parameter); ii) a shift register FIFO which does not properly add data to the last register in the pipeline; and iii) 2-5

correct circular pointer FIFOs in parallel with a non-deterministic arbiter and credit counters for managing data flow. The reset state of the credit counter has one too many credits, so data can be pushed to a full FIFO. The single FIFOs have two actions each: one for pushing data, and one for popping data. For the arbitrated circuits, there is a separate action for pushing data onto each FIFO as well as a single request action which is enabled whenever any FIFO is non-empty. There is an inherent symmetry in all of these designs. Consider any of the FIFOs. There are two main actions: pushing data (which is enabled if the FIFO is not full); and popping data (which is enabled if the FIFO is not empty). In a state where both are enabled, pushing data followed by popping results in the same state as popping and then pushing the same data. Furthermore, the actions can be performed simultaneously, but requiring that they are performed separately should not change the reachable states (depending on the implementation), so RIS is applicable.

Our experiments vary both the parameterizable data width and depth of the packet movers, by sweeping all powers of two between 2 and 128. All benchmarks contain injected bugs and reach the bug at a deep bound relative to the depth. We used a timeout of 4 hours. We use our prototype flow for checking the conditions and CoSA for bounded model checking.[6] For condition (3), we had to write one guard which is true whenever the scoreboard counter is greater than zero to handle an edge case. This same guard was used for every design, but an appropriate invariant relating the scoreboard counter to the internal state of the system being verified would also have worked. The open-source shift register FIFO required one more guard about the number of stored elements. We obtained both guards by observing counterexamples.

Table 1 compares the number of solved instances (49 total per row) within the timeout and the average runtime of commonly solved instances in seconds. Columns marked “PR” used the POR and RIS constraints. We additionally use the following abbreviations: “com” for commercial, “cp” for circular pointer, “sr” for shift register and “arb” for arbitrated. In Fig. 4 we plot the actual runtime on a log-scale for all the benchmarks with and without POR and RIS. The dotted lines show 10x and 100x improvements.

**Analysis.** There is a cluster of points in the bottom left of Fig. 4 which are solved extremely quickly by both approaches, but slightly faster without POR and RIS. These are results on benchmarks with very small parameter values, where the bug occurs at a low depth, and so the POR and RIS results are dominated by the time taken to check the conditions. However, as the parameter sizes, and runtimes, increase, it is clear that POR and RIS can result in exponential speed ups.

Recall that one concern is that RIS could extend the bound needed to reach the bug. In the shift register and arbitrated FIFO systems, it extended the bound by a few steps. However, for the bug in the open-source circular pointer FIFO, it doubled the bound needed to reach the bug. Regardless, this was more than

[6] Note: CoSA's bounded model checking performance is comparable to commercial model checkers on these benchmarks.

compensated for by the symmetry-breaking of POR, as evidenced by the faster times to reach the bug. The deepest bound was 260 which occurred at FIFO depth 129.

It is interesting to note that encoding the transition systems to SMT using the theory of arrays was always slower for bounded model checking, but was noticeably faster for checking RIS and POR conditions. Perhaps this is because the state comparison is easier for the solver to reason about using array extensionality [23].

We have demonstrated that these techniques work well for packet movers. In part, this is because packet movers are often well-constrained by their environmental assumptions, and their behavior is largely independent of incoming data values. Furthermore, we typically expect the POR and RIS conditions to hold for a correct packet-mover implementation, so a failure in a condition could identify a bug.

## 6 Related Work

Various techniques have been employed to accelerate bounded model checking. The authors of [19] use BDDs to accelerate BMC, and the techniques introduced in [35,36] exploit the structure of BMC queries to help the SAT solver. The authors of [18] take advantage of structural information with an SMT framework tailored for BMC. Our technique is similar in that we speed up bounded model checking by adding constraints to the transition system, but we obtain constraints using partial order reduction analysis.

Wang et al. [37] pioneered partial order reduction for symbolic software model checking, guaranteeing optimal reduction for two threads. Their follow-up paper, [21], extended this framework to find the optimal reduction for any number of threads. We adapted their symbolic POR technique for synchronous hardware model checking, and developed reduced instruction sets to improve the efficacy of POR in this new domain. Bhattacharya et al. used a SAT solver to directly check guarded independence conditions (as opposed to checking syntactic properties) for asynchronous rule-based languages [3]. We also check conditions directly, but in a synchronous setting.

The techniques developed by McMillan, *temporal case splitting* and *path splitting* [28], provide a framework for splitting on possible values at a given timestep. These approaches deal with system executions, but still rely on breaking data symmetries for performance. In contrast, our techniques focus on mitigating path symmetries.

The work of Bengtsson et al. [2] extended POR to timed automata using a local-time desynchronization of clocks, followed by resynchronization with an added global clock. Similarly, our techniques adapt POR by modifying the system. However, our approach targets a different domain, and only modifies the original system by adding constraints.

## 7 Conclusion

We have presented a set of conservative conditions over transition systems and automated techniques for proving these conditions. If the conditions can be proved, then constraints can be added to the system that break path symmetries. We evaluated our approach on parameterized open-source and commercial packet-mover circuits and demonstrated significant improvements in bounded model checking performance.

Some potential future work includes improvements to the **ProveRIS** procedure, investigating applications of partial order reduction to sequentially-composed packet movers, developing more targeted condition proofs by associating actions with particular data inputs, and building an interactive tool which helps the user identify and manage reduced instruction sets and partial order reductions.

## 8 Data Availability Statement

The experimental results and the necessary software for reproducing results in a standard Ubuntu 18.04 installation are available in the Figshare repository: https://doi.org/10.6084/m9.figshare.11874687.
